# *GLS* loss of function causes autosomal recessive spastic ataxia and optic atrophy

**DOI:** 10.1002/acn3.522

**Published:** 2018-01-22

**Authors:** David S. Lynch, Viorica Chelban, Jana Vandrovcova, Alan Pittman, Nicholas W. Wood, Henry Houlden

**Affiliations:** ^1^ Department of Molecular Neuroscience UCL Institute of Neurology Queen Sq London WC1N 3BG United Kingdom; ^2^ Neurogenetics Unit National Hospital for Neurology & Neurosurgery Queen Sq London WC1N 3BG United Kingdom

## Abstract

We describe a consanguineous family in which two brothers were affected by childhood onset spastic ataxia with optic atrophy and loss of motor and language skills. Through a combination of homozygosity mapping and whole‐genome sequencing, we identified a homozygous copy number variant in *GLS* as the cause. The duplication leads to complete knockout of *GLS* expression. *GLS* encodes the brain‐ and kidney‐specific enzyme glutaminase, which hydrolyzes glutamine for the production of glutamate, the most abundant central nervous system neurotransmitter. This is the first report implicating *GLS* loss of function in human disease.

## Introduction

Autosomal recessive spastic ataxias are a diverse group of neurodegenerative disorders caused by mutations in a variety of genes including *SACS,* 1 *KIF1C,* 2 *MARS2,* 3 and *AFG3L2*.^4^ These genes encode proteins with a variety of cellular functions, from the ubiquitin–proteasome system to axonal transport and protein translation.

Symptoms usually develop in childhood and include variable degrees of cerebellar ataxia and lower limb spasticity, often accompanied by optic atrophy, dystonia, and epilepsy. In this study, we describe a family affected by autosomal recessive spastic ataxia with optic atrophy, in whom whole‐exome sequencing failed to make a diagnosis. By employing a combination of whole‐genome sequencing and homozygosity mapping, we detected a homozygous duplication spanning exon 1 of the gene *GLS*, leading to complete loss of *GLS* mRNA and protein expression. This is the first time that the loss of function *GLS* mutations have been implicated in human disease.

## Materials and Methods

### Next‐generation sequencing

Whole‐exome sequencing was performed with the Agilent SureSelect All Exon kit (Agilent, California, USA) as previously described.^5^ Whole‐genome sequencing was performed by deCODE genetics. Genomic coordinates were numbered according to the hg19 build of the human genome reference.

### Duplication‐specific PCR and functional studies

Duplication‐specific PCR was carried out on gDNA using the following primers; Forward: GTCTCGCT CTGTCACACAGG, Reverse: TCACTGATAAGCCCTGCCAA.

For RT‐PCR analysis, total RNA was extracted from leukocytes, and reverse transcribed to cDNA using the High Capacity cDNA Synthesis kit (Applied Biosystems). A PCR was then performed with the following primers; Forward: CTGTCCAGCTCTCCTTCGG, Reverse: ACAGCAAATCTTCCAAGCTAGG.

For Western blotting, total proteins were extracted from cultured fibroblasts, resolved by SDS‐PAGE and transferred to PVDF membranes. For glutaminase, the following knockout validated antibody was used: Anti‐Glutaminase antibody, Abcam ab156876, which recognizes both the KGA and GAC isoforms, and for loading control, Anti‐beta Actin antibody (Abcam ab8226) was used.

## Results

### Clinical findings

We identified a family in which two brothers were affected by an aggressive form of spastic ataxia with optic atrophy. The parents of the affected patients were cousins and were from a geographically isolated region of Turkey. The inheritance pattern was autosomal recessive, and the two patients included in this study were the only affected members in the family. Initially, both boys developed normally, with an uneventful pregnancy and delivery, and reached motor and language milestones at appropriate ages. At the age of 5 years, both boys had normal motor coordination, and could run and play games. They attended school and there were no concerns with their cognitive or motor development. However, at the age of 7 years, both boys began to develop difficulty with coordination and gait. By the age of 8, they began to develop visual loss due to progressive optic atrophy. With time, the syndrome progressed into a profound ataxia with upper motor neuron signs and loss of language skills. Visual impairment progressed to perception of light only at 14 years.

At the age of 27 and 30, now both patients can stand and walk only with assistance, and are otherwise wheelchair dependent. There is optic atrophy and bilateral gaze evoked nystagmus with some limitation of upgaze. Tone is increased in the upper and lower limbs with symmetrically brisk reflexes. There is significant limb and truncal ataxia. Speech is profoundly dysarthric and language skills are limited to the repetition of single words, such as names. Sensory examination is normal.

**Figure 1:**
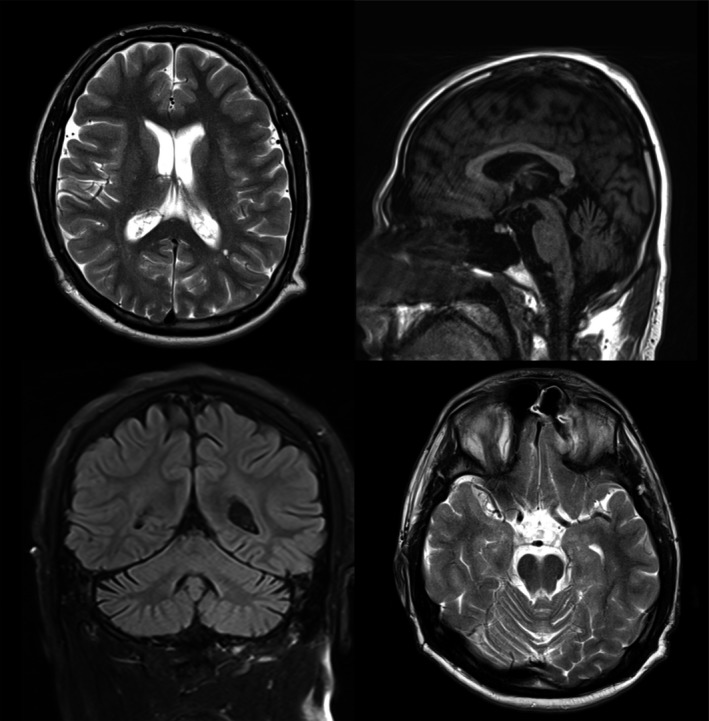
MRI imaging of Patient 1 demonstrates mild cerebellar atrophy with preservation of the cerebral and brainstem volumes and normal white matter signal.

MRI imaging in both patients demonstrated mild cerebellar atrophy with preservation of the cerebral and brainstem volumes and normal white matter signal (Fig. 1). Nerve conduction studies and muscle biopsy were normal, indicating that the syndrome is confined to the central nervous system. An extensive series of tests for childhood neurodegenerative diseases was negative including metabolic testing for Batten's Disease and Neuronal Ceroid Lipofuscinosis.

### Genetic results

Considering the pedigree and history of consanguinity, we felt that this syndrome was likely to be due to a homozygous mutation. To identify the causative mutation, we first carried out whole‐exome sequencing (WES) on both affected family members. We employed a filtering strategy to prioritize rare, homozygous variants shared between both affected patients that were likely to affect protein function. However, this strategy did not identify any potentially disease‐causing mutations. To exclude the possibility that the causative gene lay within a region of poor exome coverage, we then carried out whole‐genome sequencing (WGS) on Patient 1.

We prioritized rare homozygous variants with likely deleterious effect not contained in our in house WGS database. This also failed to identify any likely pathogenic variants. To narrow the disease locus, we carried out genome wide genotyping on all available family members using the Illumina CytoSNP12 array, and used HomozygosityMapper^6^ to identify shared regions of homozygosity in affected patients.

**Figure 2:**
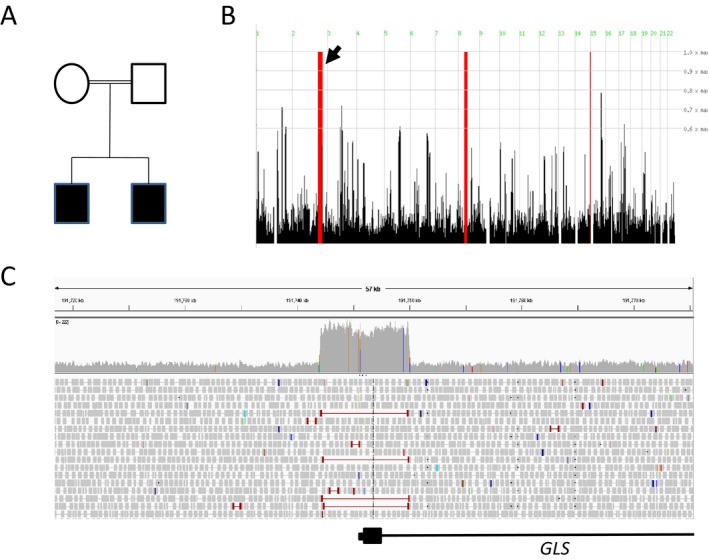
Family pedigree is shown (A), where filled boxes indicate affected patients. Homozygosity mapping identified three regions of significant homozygosity on Chromosomes 2, 8, and 14 (B). Whole‐genome sequencing identified an approximately 8 Kb region with significantly increased read depth and split‐paired reads spanning exon 1 of the gene *GLS,* indicating a duplication (C).

This identified 3 shared homozygous regions present in both siblings (Fig. 2B). With the hypothesis that the disease‐causing mutation could be a copy number variant or structural rearrangement, we used the software program LUMPY,^7^ to analyze the WGS data on Patient 1. We limited our analysis to CNVs found within the linked homozygous regions that overlapped with the coding region of a gene, and were therefore likely to have a functional outcome. LUMPY identified only one such variant, an 8 kb duplication spanning exon 1 of the gene *GLS*. A large increase in read depth and split reads mapping to the duplication breakpoint were visible in IGV (Fig. 2C). The breakpoints of the duplication were predicted to lie within AluSx SINE elements, which are approximately 90% homologous, implying the mechanism of duplication as nonallelic homologous recombination. At an RNA level, a duplication of exon 1 would lead to a premature stop codon with likely nonsense mediated decay (NMD) of the affected transcript.

**Figure 3:**
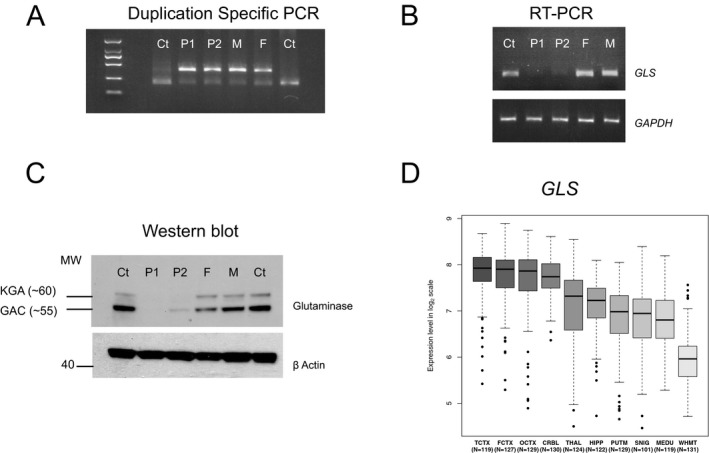
After duplication‐specific PCR, a band is visible in the parents, and affected offspring, but not in controls (A). Reverse transcription PCR showed absent or severely reduced *GLS* RNA compared to control or to the parents (B). Reduced glutaminase protein levels were confirmed by Western blotting (C). GLS is highly expressed in brain from early development, and is widely expressed in cerebellum and cortex in data from the BRAINEAC consortium (D).

To confirm the presence of the duplication and map its breakpoint more precisely, duplication‐specific PCR was performed. In both children and parents, duplication‐specific PCR revealed an aberrant band and sequencing confirmed the breakpoint to have occurred between a highly homologous region of Intron 1‐2 (Chr 2: 191750021) and the 5′ untranslated region (Chr2: 191742079) (Fig. 3A).

To characterize the effect of the duplication on RNA, we extracted total RNA from peripheral blood leukocytes and reverse transcribed it to cDNA. In the affected patients, *GLS* cDNA was either not present or was severely reduced. A control RT‐PCR for *GAPDH* was used to confirm the presence of intact cDNA in all samples (Fig. 3B). We concluded that the homozygous duplication leads to complete knockout of GLS expression in patient tissue. This was confirmed by western blotting using a glutaminase antibody on whole‐cell lysates extracted from fibroblast cell lines of the patients, their parents, and two unrelated controls (Fig. 3C).

## Discussion

*GLS* encodes the brain‐ and kidney‐specific enzyme glutaminase, which hydrolyzes glutamine for the production of glutamate. Since neurons cannot synthesize glutamate de novo from glucose, they depend heavily on the recycling of glutamine and glutamate between neurons and astrocytes for neurotransmission.

This occurs when glutamate at the synapse is imported into astrocytes and converted to glutamine. This glutamine diffuses into the extracellular space and is take up by neuron presynaptic terminals, where glutaminase converts it to glutamate. The glutamate is then packaged into synaptic vesicles for release.

In keeping with its essential role in the glutamate pathway, *GLS* is widely expressed in all brain regions from early development, with highest levels found in the cerebral cortex and cerebellum (Fig. 3D, from the BRAINEAC database^8^). The gene is intolerant to loss of function mutations in humans, with a pLi score^9^ of 1.

Interestingly, *GLS* ‐/‐ mice die shortly after birth.^10^ Cultured neurons from the mice show a reduction in depolarization‐evoked glutamate release and altered excitatory postsynaptic potentials (EPSCs). In addition, it has been shown that knockdown of *GLS* in human neural progenitor cells (NPCs) leads to a significant reduction in NPC survival and ability to differentiate into neurons.^11^ Taken together, these findings would predict that complete loss of *GLS* expression would not be compatible with human growth and development. However, we clearly show that *GLS* is not absolutely required for early human development, although complete absence of *GLS* activity leads to a complex phenotype with aggressive, childhood onset spastic ataxia, and optic atrophy. The lack of significant brain atrophy on imaging, and the prominent involvement of the optic nerves, for which glutamate is the primary excitatory neurotransmitter, supports our findings that the syndrome described here is primarily due to a functional failure of neurotransmission, rather than an alternative neurodegenerative process.

This report raises important questions about the mechanism of glutamate recycling and neurotransmission in humans. We hypothesize that survival is due to a compensatory mechanism, either through increased expression of *GLS2* isoforms in the CNS, or through an alternative process to generate glutamate, such as from *α*‐ketoglutarate by the enzyme alanine transaminase. This report also adds to the number of synaptic genes implicated in neurological diseases, including in spastic ataxias, such as *VAMP1*.

In summary, through a combination of whole‐genome sequencing, homozygosity mapping and functional studies, we show that *GLS* knockout is compatible with human life, and causes childhood onset spastic ataxia and optic atrophy.

## Author Contributions

David Lynch performed conception and design of the study, interpretation of data, and drafting of the manuscript. Viorica Chelban, Jana Vandrovcova, and Alan Pittman involved in collection and interpretation of data and drafting of the manuscript. Nicholas W Wood and Henry Houlden involved in conception and design of the study and critical review of the manuscript.

## Conflicts of Interest

Dr Lynch, Dr Chelban, Dr Vandrovcova, Dr Pittman, Prof Houlden, and Prof Wood have nothing to disclose.
